# How Noisy is Lexical Decision?

**DOI:** 10.3389/fpsyg.2012.00348

**Published:** 2012-09-24

**Authors:** Kevin Diependaele, Marc Brysbaert, Peter Neri

**Affiliations:** ^1^Ghent UniversityGhent, Belgium; ^2^University of AberdeenAberdeen, UK

**Keywords:** internal noise, lexical decision, signal detection, megastudies, lexicon projects

## Abstract

Lexical decision is one of the most frequently used tasks in word recognition research. Theoretical conclusions are typically derived from a linear model on the reaction times (RTs) of correct word trials only (e.g., linear regression and ANOVA). Although these models estimate random measurement error for RTs, considering only correct trials implicitly assumes that word/non-word categorizations are without noise: words receive a yes-response because they have been recognized, and they receive a no-response when they are not known. Hence, when participants are presented with the same stimuli on two separate occasions, they are expected to give the same response. We demonstrate that this not true and that responses in a lexical decision task suffer from inconsistency in participants’ response choice, meaning that RTs of “correct” word responses include RTs of trials on which participants did not recognize the stimulus. We obtained estimates of this internal noise using established methods from sensory psychophysics (Burgess and Colborne, 1988). The results show similar noise values as in typical psychophysical signal detection experiments when sensitivity and response bias are taken into account (Neri, 2010). These estimates imply that, with an optimal choice model, only 83–91% of the response choices can be explained (i.e., can be used to derive theoretical conclusions). For word responses, word frequencies below 10 per million yield alarmingly low percentages of consistent responses (near 50%). The same analysis can be applied to RTs, yielding noise estimates about three times higher. Correspondingly, the estimated amount of consistent trial-level variance in RTs is only 8%. These figures are especially relevant given the recent popularity of trial-level lexical decision models using the linear mixed-effects approach (e.g., Baayen et al., 2008).

## Introduction

Word recognition research often makes use of the lexical decision task (LDT). In this task participants are presented with strings of letters and have to decide whether the letters form an existing word (e.g., BRAIN) or not (BRANK). The main dependent variable is the decision time of the correct yes-responses to the word trials. A secondary variable is the decision accuracy. Originally it was thought that lexical decision performance was a pure measure of lexical access (i.e., the time needed to activate individual word representations in the mental lexicon; see Balota and Chumbley, 1984, for references to this literature). Later it became accepted that lexical decision times are also affected by the similarity of the presented word to the other words of the language (i.e., the total activation in the mental lexicon, usually defined as the number of words that can be formed by replacing a single letter of the original word; Grainger and Jacobs, 1996) and the degree of similarity between the word and non-word stimuli (Gibbs and Van Orden, 1998; Keuleers and Brysbaert, 2011).

The primacy given to reaction times (RTs) over decision accuracies reflects the fact that language researchers are primarily interested in the speed of word recognition rather than the precision of the process (given that in normal reading next to all words are recognized). In the vast majority of studies, RTs of correct word responses are modeled as a linear combination of a few fixed predictor variables and random measurement error. The estimate of the latter component represents the expected RT fluctuation with respect to repeated sampling (i.e., to what degree RTs can be expected to vary in a replication of the experiment). However, when one estimates fixed and random effects for RTs in this way, it is assumed that the response level is fixed and thus will not vary across different replications of the same experiment. Participants respond “yes” because they have recognized the word, and they respond “no” to those words they do not know. In other words, a correct response is assumed to be fully reliable with respect to repeated sampling. To ensure valid RTs, participants and word stimuli are selected in such a way that overall performance accuracy is higher than 80–90% (that is, the words are selected so that they are known to most of the participants).

Thus, statistical models of lexical decision experiments typically take measurement error into account with respect to decision times, but they assume this error to be zero for the actual decision itself. This notion, which is routinely adopted in lexical decision research, does not take into full account an established result in psychophysical research, namely that a large part of the variance in individual response choice reflects internal cognitive noise. Because of this noise, measurements of both response time and response choice vary to some extent when individuals respond to the same stimuli on repeated occasions. Psychophysicists investigate this source of noise by examining the probability distribution of responses to a particular stimulus rather than assuming that each response is a veridical, fixed estimate of stimulus processing difficulty. When fitting models to predict an individual’s “correct” behavior, they then accept that the success of doing so depends on the amount of internal noise or internal consistency, which limits the amount of variance one can aim to explain. For a long time, psycholinguists have avoided the issue of internal noise by averaging data across a number of different experimental trials, which leads to analysis-units (i.e., means) with smaller standard errors, but the issue is becoming increasingly relevant as more and more researchers are beginning to examine RT distributions instead of point estimates (e.g., Yap et al., 2012) and are using statistical analyses based on individual trials instead of aggregated ones (e.g., Baayen et al., 2008).

One solution for lexical decision research could be to perform the data-analysis with mathematical models that, for a given trial, predict the RT, and response choice, including estimates for both RT- and response level measurement errors. Unfortunately, such models (e.g., Ratcliff et al., 2004) are currently not as developed as the linear framework, meaning that they do not yet provide ready estimates for multiple fixed effects and multi-level random structures with reasonably scaled data sets (e.g., Pinheiro and Bates, 2000; Rigby and Stasinopoulos, 2005).

Another reason why the linear framework is popular is that no-one knows how large the internal noise is and, therefore, to what extent the assumption of fixed responses is unwarranted. Most researchers will acknowledge that assuming zero-measurement error for response categories is most likely wrong, but a formal analysis of the degree of internal noise in a LDT is lacking. To fill this gap, in the present manuscript we opt for a general approach borrowed from the psychophysical literature (Burgess and Colborne, 1988; Ahumada, 2002; Neri, 2010). In this line of research, participants are asked on each trial to discriminate a signal + noise stimulus (e.g., a target letter embedded in unstructured information) from a noise-alone stimulus (i.e., the unstructured information alone). In the first half of the experiment each trial presents new information; we refer to this part as the “first pass.” In the second half, the stimuli of the first pass are repeated (albeit often in a different order) and participants have to respond to them again; we refer to this part as the “second pass.” The inclusion of two passes with the same information allows researchers to compute two quantities: the percentage of trials on which the observer responded correctly (i.e., correctly identified the signal; defined as ρ), and the percentage of trials on which the observer gave the same response to a given trial on both the first and the second pass (defined as α). Burgess and Colborne (1988), Ahumada (2002), and Neri (2010) outlined how these two quantities can be used to estimate the amount of internal noise associated with the observers’ stimulus processing.

The model developed by Burgess and Colborne (1988), Ahumada (2002), and Neri (2010) represents a variant of standard signal detection theory (SDT, Green and Swets, 1966). In this model internal responses to external stimuli are assumed not only to reflect external noise (i.e., noise associated with the stimulus and having standard deviation σ_*N*_), but also internal processing noise (defined as σ_*I*_). Specifically, internal responses to noise (*r*_*N*_) and signal + noise stimuli (*r*_*S+N*_) are modeled as follows:

$$\begin{array}{llll} r_{N} & \sim \mu_{N}+N\left(0,\sigma_{N}\right)+N\left(0,\sigma_{1}\right) & & \text{(1)}\\ r_{S+N} & \sim S+\mu_{N}+N\left(0,\sigma_{N}\right)+N\left(0,\sigma_{1}\right) & & \text{(2)} \end{array}$$

As a result, the internal responses to external noise are assumed to be normally distributed with mean μ_*N*_ and standard deviation determined by both σ_*N*_ (external noise) and σ_1_ (internal noise). On signal + noise trials a fixed internal value *S* is added.

The contribution of external noise in Eqs 1 and 2 can be neutralized by normalizing both equations with respect to external noise (this is done by subtracting μ_*N*_ and dividing the outcome by σ_*N*_, as for the calculation of z-scores). The outcome gives:

$$\begin{array}{llll} r_{N}^{\prime } & \sim N\left(0,1\right)+N\left(0,\gamma \right) & & \text{(3)}\\ r_{S+N}^{\prime } & \sim d_{\text{in}}^{\prime }+N\left(0,1\right)+N\left(0,\gamma \right) & & \text{(4)} \end{array}$$

In these equations, the internal noise and the internal signal strength are expressed in units of external noise $\left(\gamma =\frac{\sigma_{1}}{\sigma_{N}},d_{\text{in}}^{\prime }=\frac{S}{\sigma_{N}}\right)$. The normalized internal signal strength $\left(d_{\text{in}}^{\prime }\right)$ is called the signal detectability index or input sensitivity.

Burgess and Colborne (1988) showed how the parameters in Eqs 3 and 4 can be derived from the values of ρ and α in a double-pass design. More specifically, they showed that good estimates for $d_{\text{in}}^{\prime }$ and σ_*I*_ can be obtained through minimizing the mean-square error between the predicted and observed values for ρ and α (see also below).

Neri (2010) observed that the internal noise across a wide range of perceptual tasks followed a lognormal distribution with γ = 1.35 ± 0.75 SD. The fact that the internal noise exceeded the external noise (i.e., γ > 1) was surprising, as it suggested that psychophysical choice is affected more by internal perturbations than by external variation. Indeed, in the first study, Burgess and Colborne (1988) expected less internal noise than external noise and obtained a ratio of γ = 0.75 ± 0.1 SD. Neri (2010) explained the discrepancy between his finding and Burgess and Colborne’s by pointing out that the experiments in his review included more complex tasks than the one used by Burgess and Colborne. Indeed, when Neri (2010) restricted his analysis to low-level perceptual tasks, he obtained a value of 0.8 consistent with the earlier study of Burgess and Colborne. With more complex tasks, however, he observed γ-values larger than one.

These results from the psychophysical literature may have important implications for the LDT. Given that this is a complex task, one may expect larger internal noise than external noise, meaning that participants are rather inconsistent in their responses, answering “non-word” to stimuli in the second pass that received a “word” response in the first pass (and vice versa). If the findings from the psychophysical literature (Green, 1964; Burgess and Colborne, 1988; Neri, 2010) generalize, then we can expect that only 70 to 84% of the lexical decisions will be consistent. This means that, even if we had access to the best possible model of how participants operate, we would be able to predict only about three quarters of the trial-level data. Such a finding would clearly be at odds with the implicit assumption in psycholinguistics that a yes-response to a word trial can be interpreted as evidence that the person knows the word and has recognized it (i.e., that the response choice is error-free).

On the other hand, some features of a typical LDT may make it more robust against the degree of inconsistency reported for complex psychophysical tasks (Neri, 2010). A potentially relevant factor in this respect is that in a typical lexical decision experiment stimuli are shown until the participants respond (usually for a maximum of 1500 ms). This mostly results in percentages of correct responses (averaged across easy and difficult words/non-words) of more than ρ = 0.9. The LDT protocol differs from customary practice in SDT experiments, where the signal-to-noise ratio of the stimulus is selected to target a threshold output sensitivity of *d*′ = 1 (Green and Swets, 1966)^1^. Indeed, in the experiments surveyed by Neri (2010) the average accuracy was about ρ = 0.75 (observers responded correctly on three out of four trials), which corresponds to a *d*′-value close to unity (following the equation $d^{\prime }=\sqrt{2}\Phi^{-1}\left(\rho \right),$ where Φ is the cumulative standard normal distribution function). It is not inconceivable that response consistency is higher for clearly visible stimuli than for briefly presented stimuli, which may result in lower internal noise values for LDT than psychophysical tasks of comparable complexity. Although this argument seems plausible, it would be better, of course, if it were based on explicit empirical testing rather than on a tacit assumption. Hence, the present experiment.

In addition, the psychophysical approach introduced by Burgess and Colborne (1988) and Neri (2010) can be extended to RTs. Although this kind of analysis has not been reported before and is not established in the psychophysical literature, there are no *a priori* theoretical objections precluding it. All that is needed is a situation in which participants respond twice to a sufficiently large sequence of words and non-words. The new analysis is interesting because it only assumes that each RT value is the sum of two components (one stimulus-dependent and one person-related). There is no need to make further assumptions about the distribution of the components (see Materials and Methods), so that the approach is extremely general, encompassing all models based on a quasi addition of stimulus-dependent and person-related variability.

In summary, we will apply the psychophysical analysis method introduced by Burgess and Colborne (1988) and Neri (2010) to the LDT. This will allow us (1) to find out to what extent the implicit psycholinguistic assumption of error-free word- and non-word responses is warranted, and (2) to determine the degree of consistent trial-level variance that can be explained in RTs. To foreshadow our findings, we will observe that the contribution of internal noise to lexical decision is much larger than commonly assumed. This is particularly the case for response-selection to low-frequency words and for RTs.

## Materials and Methods

Data were obtained from the Dutch Lexicon Project (Keuleers et al., 2010b). In this study, 39 participants responded to 14,339 word trials and 14,339 non-word trials, which were presented in 58 blocks of 500 stimuli (the last block was shorter). Participants responded with their dominant hand when they thought a word was presented, and with their non-dominant hand otherwise. Importantly, to gage practice effects in the study, the sequence of stimuli in block 50 was identical to that of block 1. As participants could only finish four blocks in an hour and rarely did more than six blocks each day, for most participants there were several weeks (and over 20 K lexical decision trials) between the first and the second run. In this way, the results were unlikely to be influenced by repetition priming effects and other influences due to episodic memory. Indeed, Keuleers et al., 2010b, Figure 1) found that the increase in response speed and accuracy across block 1 and 50 was very modest. For word responses participants were on average 35 ms faster and 5% more accurate. For non-words there was a 22 ms decrease in response speed, but accuracy was 2% worse in block 50. Because participants got different permutations of the complete stimulus list, the words each one saw in blocks 1 and 50 were a unique subsample of the stimulus list. As a result, the analyses presented below are not limited to a particular section of the stimulus list (which would have been the case if the words had been the same for every participant). Therefore, the characteristics of the stimuli are the same as those of the Dutch Lexicon Project as a whole (see Keuleers et al., 2010b, Table 1, for a summary and a comparison with the lexicon projects in other languages).

**Figure 1:**
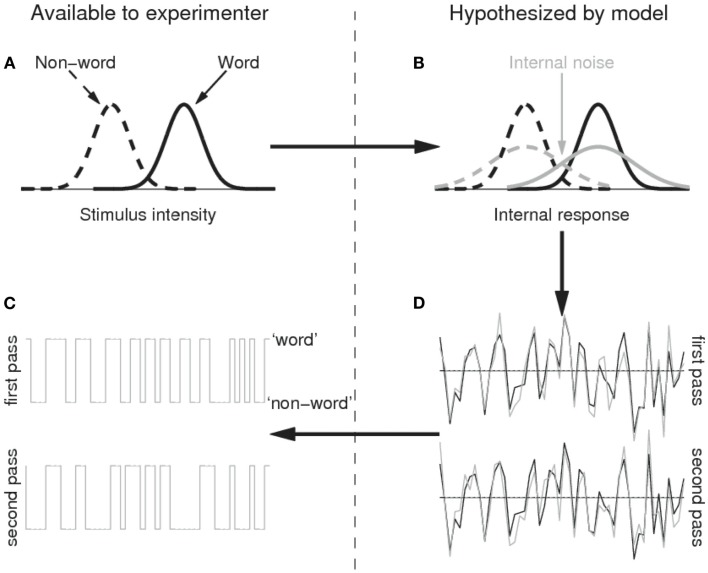
**Illustration of the SDT model with internal noise applied to the lexical decision task**. Word and non-word stimuli map onto the stimulus intensity/internal response dimension with the same normal variance, but with different means (i.e., the word stimuli are on average more word-likely than the non-word stimuli; **(A)** because of the internal noise source **(B)**, however, the internal responses to the same stimuli differ to some extent between repetitions **(D)**; the *x* axis represents time, the *y* axis the internal response: e.g., up is evidence toward a word response, down is evidence toward a non-word response) and the word/non-word responses will not be fully consistent **(C)**. Please refer to Methods for further details.

Further of importance for the present analyses is that participants were not allowed to drop consistently below 85% overall accuracy (otherwise they were asked to leave and did not receive the full financial reimbursement). Such accuracy requirements are standard in lexical decision, where the data of participants with, for example, more than 20% errors are discarded. Accuracy was higher for non-words (94%) than for words (84%), as can be expected from the fact that not all words were known to the participants (some had very low frequencies of occurrence).

The LDT conforms to a yes-no design (Green and Swets, 1966): a word (target) or a non-word (non-target) is presented on every trial, and the participant is asked to choose between these two possibilities. So, the analysis proposed by Burgess and Colborne (1988) can be applied. A complicating factor, however, is that the equations outlined by Burgess and Colborne require the absence of response bias (i.e., participants are not more likely to select one response than the other). In the Dutch Lexicon Project, there was a small response bias toward non-word responses (−0.31), which was statistically significant [*t*(38) = −10.46, *p* < 0.001]. Luckily, Ahumada (2002, Eqs 3.1.6 and 3.1.7) derived the equations needed to estimate internal noise under conditions of potential bias. These are (the reader is referred to the original publication for details on how the equations were derived):

$$\begin{array}{llll} p_{\left[0,0\right]}^{*} & =\overset{\infty }{\underset{-\infty }{\int }}\Phi {\left(\frac{\Phi^{-1}\left(p_{\left[0,0\right]}\right)\sqrt{1+\gamma^{2}}-x}{\gamma }\right)}^{2}\phi \;\left(x\right)\;\text{dx} & & \text{(5)}\\ p_{\left[1,1\right]}^{*} & =\overset{\infty }{\underset{-\infty }{\int }}{\left[\text{1}-\Phi \left(\frac{\Phi^{-1}\left(p_{\left[1,0\right]}\right)\sqrt{1+\gamma^{2}}-x}{\gamma }\right)\right]}^{2}\phi \;\left(x\right)\;\text{dx} & & \text{(6)} \end{array}$$

In these equations the following notation is used: *p*_[s,r]_ is the proportion of trials on which the observer responded *r* (0 for non-word, 1 for word) when presented with stimulus *S* (0 for non-word, 1 for word); $p_{\text{[s,r]}}^{*}$ is the proportion of trials on which the observer responded *r* to both passes of stimulus *S*; Φ is the cumulative standard normal distribution function; ϕ is the standard normal distribution density function; γ is the standard deviation of the internal noise source in units of the external noise standard deviation $\left(\text{i}\text{.e}\text{.,}\;\frac{\sigma_{I}}{\sigma_{N}}\right).$

For non-mathematical readers, it may be good at this moment to flesh out the model to some extent. The model basically assumes that there are two stimulus categories (words and non-words), which map onto a single quantity, which can be called “the degree of wordness” (the *x* axis of Figure 1A). The distribution of stimuli belonging to the word category is assumed to have a higher mean value of wordness than the non-word category, but to have the same standard deviation (Figure 1A). Because of the variability in each category, the wordness distributions of both categories partly overlap (i.e., some non-words have a higher degree of wordness than some words). The variability introduced at this stage is called external noise, because it is driven by the external stimulus (the degree of wordness each word and non-word in the experiment has).

The model further assumes that the wordness intensity of a stimulus is mapped onto a corresponding quantity within the observer’s brain, which preserves the original structure of the input (the black lines in Figure 1B). However, the output of this mapping is not error-free due to internal noise. As a result, the variability of the quantities in the observer’s brain is larger than the variability of the stimulus intensity levels (the gray lines in Figure 1B). This is true as much for words as for non-words. Furthermore, the variability introduced by the internal noise source is decoupled from the stimulus, so that the output of the internal representation in response to two presentations of the same stimulus need not be the same. As a result, the internal responses to a given sequence of stimuli will contain the repetitive structure present in the stimulus sequence (due to the degree of wordness of each stimulus; black traces in Figure 1D), but in addition it will contain some non-repetitive structure due to the variability introduced by the internal noise (the gray traces in Figure 1D). Finally, the SDT model assumes that observers set a threshold value for converting the output from the internal representation into a binary response of the word/non-word type. If the internal representation exceeds this threshold (indicated by horizontal line in Figure 1D) they respond ‘word’, otherwise they respond “non-word.”

From the response sequences in the first and the second pass we can compute the quantities needed for Eqs 5 and 6, namely *p*_[0,0]_ and *p*_[1,0]_, $p_{\text{[0,0]}}^{*}$ and $p_{\text{[1,1]}}^{*}$. On the basis of these quantities, we can then estimate the internal noise intensity (γ) that minimizes the mean-square-error between predicted and observed $p_{\text{[0,0]}}^{*}$ and $p_{\text{[1,1]}}^{*}$ given *p*_[0,0]_ and *p*_[1,0]_. If the sequence of responses in the first and the second pass is exactly the same, the best estimate of γ will be 0, because there is no internal noise (the responses are fully driven by the wordness values of the stimuli). Conversely, the more the sequences of responses differ between first and second pass, the higher the estimated γ-value must be to account for the absence of consistency.

To estimate the degree of internal noise in RTs, we simply assumed that the observed RTs were the sum of two processes, one related to the stimulus and one decoupled (i.e., independent) from the stimulus. The former can be thought of as the stimulus-induced internal representation in Figure 1B (black trace); the latter as the participant-dependent internal noise (gray trace). The predicted pattern of RTs then is the same as in Figure 1D: RTs are assumed to consist of a component identical in both passes (black traces), together with a component differing between the two passes (gray traces). It is easy to show that the correlation coefficient *R* between the two sequences of RTs then equals $\frac{\sigma_{N}^{2}}{\sigma_{I}^{2}+\sigma_{N}^{2}}$, where σ_*I*_ is the standard deviation of the internal noise source and σ_*N*_ the standard deviation of the external stimulus. The quantity we are interested in is the ratio $\gamma =\frac{\sigma_{I}}{\sigma_{N}},$ i.e., the intensity of the internal noise source in units of standard deviation in the degrees of wordness. This is easily obtained by $\sqrt{\left(\frac{1}{R}-1\right).}$ Before calculating *R*, we inverse transformed all RTs (i.e., −1000/RT) to correct for the positive skew in the RT distribution (Ratcliff, 1993). Finally, we calculated the output sensitivity $d^{\prime }=\Phi^{-1}\left(p_{\left[1,1\right]}\right)-\Phi^{-1}\left(p_{\left[0,1\right]}\right)$ for each participant.

## Results

**Figure 2:**
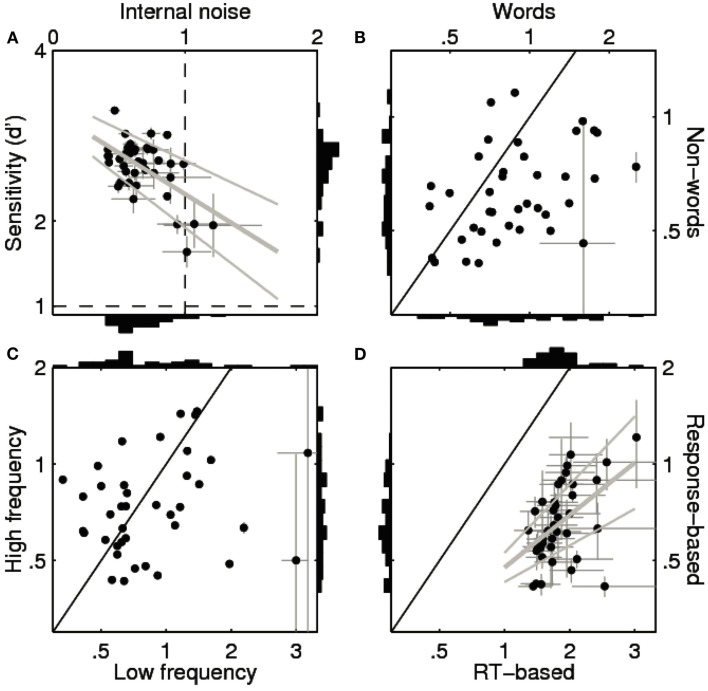
**Sensitivity and internal noise values for all 39 participants in the Dutch Lexicon Project (Keuleers et al., 2010b)**. **(A–C)** show estimates for the response choice data. **(A)** Plots internal noise (*x* axis) against sensitivity (*y* axis) across participants (one data point per participant) computed from all trials. **(B)** Plots internal noise computed from word trials only (*x* axis) against internal noise from non-word trials only (*y* axis). **(C)** Plots internal noise computed from low-frequency word trials only (*x* axis) against internal noise from high-frequency word trials only (*y* axis). Solid black lines mark identity. Error bars show ± 1 SE (smaller than symbol when not visible). **(A,D)** Include best linear fit (thick gray line) ± 1 SE (thin gray lines). **(D)** Shows internal noise computed from reaction time measurements (*x* axis) against internal noise computed from response choice data^2^ (*y* axis).

Our main findings are summarized in Figure 2. Starting with Figure 2A, we notice that sensitivity (*d*′) is considerably higher (at a value of about 2) in the lexical decision experiment than the value of 1 typically targeted in SDT experiments (Neri, 2010). This is not surprising given that SDT experiments emphasize threshold visibility, whereas lexical decision experiments emphasize clear visibility of the stimulus. A more interesting feature of Figure 2A is that the internal noise estimates (*x* axis), expressed as $\gamma =\frac{\sigma_{I}}{\sigma_{N}},$ are below 1 for nearly all participants (typically around 0.6), indicating that internal noise (σ_*I*_) was smaller than external noise (σ_*N*_). As indicated in the Introduction, this relatively low estimate is to be contrasted with the average value of 1.3 reported by Neri (2010) for complex psychophysical tasks. It therefore appears that, despite the taxing cognitive demands associated with LDT, internal noise in a lexical decision experiment is relatively low and does not exceed the external noise source (i.e., γ < 1). At the same time, the impact of internal noise is not zero, as assumed by psycholinguists. There is some degree of inconsistency in the response selections made by the participants in the first and the second pass. Not all stimuli that were “recognized” as words in the first pass were also “recognized” in the second. Similarly, not all stimuli that failed to elicit a word response in the first pass were considered as non-words in the second pass.

Further interesting is the observation that sensitivity correlated negatively with γ across individuals [*R* = −0.57, |*t*(37)| = 4.18, *p* < 0.001]. So, the most accurate participants showed the smallest γ-values (Figure 2A). This is in line with the hypothesis that the low degree of internal noise we observed in the LDT was partly due to the fact that only participants with good knowledge of the words were included in the study. Indeed, if we extrapolate the linear regression line between sensitivity and γ to *d*′ = 1, the predicted value of γ falls within the range reported for sensory processing (i.e., 1.35 ± 0.75 SD, see Neri, 2010), suggesting that the degree of internal noise in lexical decision is comparable to non-verbal perceptual tasks when task difficulty is matched.

Figure 2B shows that the internal noise is higher for words than for non-words [most of the points fall below the solid unity line; *M* = 0.32, |*t*(38)| = 4.45, *p *< 0.001]. This is in line with the observation that accuracy was higher for non-words than for words (see above). A difference in accuracy is also the most likely explanation for why internal noise was higher for low-frequency words than for high-frequency words [*M *= 0.27, |*t*(38)| = 1.91, *p *< 0.05; Figure 1C]. Participants were less accurate on trials with words that had a frequency of less than 1 occurrence per million words than on trials with higher-frequency words, and for these words they showed higher γ-values. In other words, internal noise shows a tendency to scale inversely with accuracy (non-words < high-frequency words < low-frequency words). Noise estimates are highest with low-frequency words and fall within the range reported for perceptual tasks (Neri, 2010; 1.05 ± 0.88 SD vs. 1.35 ± 0.75 SD). We also observed a significant positive correlation between the internal noise values on word and non-word trials [*R *= 0.35, |*t*(37)| = 2.28, *p *< 0.05], but not between internal noise values for high- and low-frequency words [|*t*(37)| < 1].

Finally, Figure 2D shows that γ was much higher for RTs than for response choice [*M *= 1.19, |*t*(37)| = 15.34, *p *< 0.001] with a significant positive correlation between the two estimates [*R *= 0.54, |*t*(36)| = 3.85, *p *< 0.001]. More specifically, γ was about three times higher for RTs (values around 1.8) than for response choices (values around 0.6). When estimated from RT data, none of the participants showed lower internal noise than external noise (i.e., all γ > 1). Further analyses indicated that there were no significant differences or correlations for RT-based internal noise as a function of lexicality (word|non-word) or word frequency $\left(x<1\text{ vs }x\geq 1\;\text{per}\;\text{million}\right)$.

It might be objected that all of the above-detailed measurements rely on a comparison between only two passes of the same set of stimuli. Two questions naturally arise in relation to this approach. First, are the internal noise estimates biased for low number of passes, i.e., is it expected that lower estimates may be obtained with a multi-pass procedure that employs >2 passes? Second, if the estimates are not biased, what is their precision? In relation to the former question, there is no *a priori* reason to expect that estimates should be biased depending on the number of passes involved; in support of this notion, multi-pass methods with >2 passes have reported internal noise estimates that are within the same range reported with double-pass methods (Li et al., 2006). With relation to the latter question, recent work (Hasan et al., 2012) has estimated the precision of the double-pass method to be in the range of 10–20% depending on the number of trials and observers associated with the measurements. The conclusions we draw in this article are valid within a range of error that is well within the above precision value.

## Discussion

Researchers using LDTs are typically making theoretical claims on the basis of correct word trial RTs only. The linear statistical models adopted in these studies assume random measurement error for RTs, but not for response choices. It is also not taken into account to what degree random RT fluctuations reflect participant-internal (i.e., cognitive) or merely external noise. The fact that these models assume that the actual choice for a word/non-word response is fixed, i.e., the product of an error-free system, is potentially problematic toward valid theoretical conclusions. Decisions are supposed to be 100% reliable: participants respond “yes” because they have recognized the word, and they respond “no” to the stimuli they do not know. This notion stands in sharp contrast with results from psychophysical research showing that internal noise introduces considerable inconsistency across identical trials (Burgess and Colborne, 1988).

Our goal in this study was to bridge the gap between lexical and psychophysical research traditions by analyzing the data of a recently collected, large-scale lexical decision experiment using statistical techniques based on SDT (Burgess and Colborne, 1988; Ahumada, 2002; Neri, 2010). We profited from the fact that the first block of 500 trials in the Dutch Lexicon Project (Keuleers et al., 2010b) was repeated in block 50, allowing us to measure the consistency of word/non-word choices and RTs to the same stimuli; we then used these measurements to derive corresponding internal noise estimates. Our analyses clearly document that the assumption of a noiseless decision process in the LDT is unwarranted. The amount of internal noise was substantial, not only with respect to RTs but also when computed from word/non-word choice data.

The most prominent implication of this result is that the ability to model trial-level lexical decision data is in fact more limited than is perhaps appreciated by most researchers in this field. According to our analysis, when participants are presented with the same lexical decision trials on different occasions, they will produce the same word/non-word choice on only about 83% of the trials (9% SD). This implies that an optimal choice model (i.e., a model that faithfully replicates the cognitive process used by the participant) would only be able to predict in-between 83 and 91% of the observed responses^3^ (Neri and Levi, 2006). The situation is even worse for RTs. The ratio of internal vs. external noise was considerably larger (about three times) for RTs than for the choice data (see Figure 2D). From the squared correlation across the two blocks we learn that only about 8% of the variance in the (correct and consistent) RTs was replicated (*R^2^ *= 0.08 ± 0.04 SD)^4^ prompting us to maintain modest expectations about our ability to predict trial-level RT data via, for instance, linear mixed-effects models (e.g., Baayen et al., 2008) or explicit computational models (e.g., Balota and Spieler, 1998; Seidenberg and Plaut, 1998)^5^.

**Figure 3:**
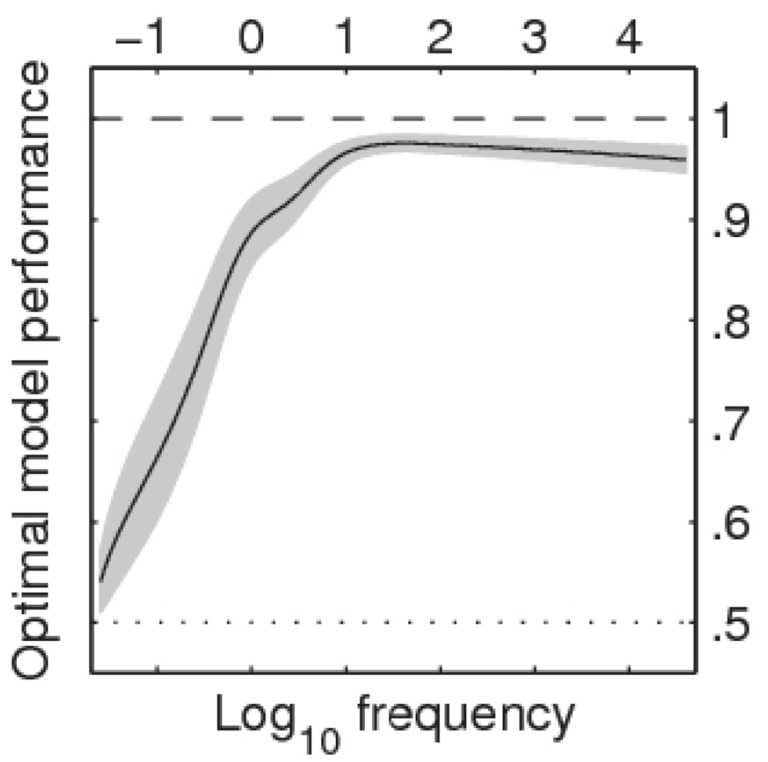
**Estimated range for optimal model performance as a 6-knot restricted cubic spline function of log-word frequency per million words (Keuleers et al., 2010a)**. Horizontal dotted line corresponds to chance level agreement.

Our analyses point to another issue. Based on the measured percentages of response choice agreement, internal noise was significantly larger for words than for non-words (Figure 2B). There was 90% agreement for non-word trials, compared to only 76% for words. The relatively poor agreement for words appears to be due to the low frequency words. This can be seen clearly when we predict the trial-level agreement data on the basis of (logarithmic) word frequency values through a mixed model with a logistic link function where participants and stimuli are used as crossed random factors. Figure 3 shows estimates for lower and upper bounds of the optimal model performance (Neri and Levi, 2006) as a function of word frequency (to model the non-linearity frequencies were expanded into natural splines). The graph illustrates that optimal performance is quite high (and similar to non-words at 90–95%) for frequencies above 10 per million, but drops to near 50% (chance) for the words with the lowest frequencies.

The observation of high accuracy for most non-words and high-frequency words, together with decreasing accuracy for low-frequency words is in line with a SDT model containing two response criteria (Krueger, 1978; Balota and Chumbley, 1984). In such a model, a low criterion is placed at the low end of the higher distribution of stimulus intensities (i.e., at the low end of the wordness values of the words in Figure 1A), and a high criterion is placed at the high end of the lower distribution (i.e., at the high end of the wordness values of the non-words in Figure 1A). Stimuli with wordness values below the low criterion elicit fast non-word responses, because virtually no words have such low values. Similarly, stimuli with wordness values above the high criterion get fast word responses, because there are virtually no non-words with such high values. Stimuli with intensity values between the low and the high criterion (for which it is not immediately clear which decision to make) get further verification processing or elicit a random response. Interestingly, the frequency value of 10 per million is the value below which the bulk of the RT word frequency effect in the Dutch Lexicon Project is situated (Keuleers et al., 2010b). This agrees with Balota and Chumbley’s (1984) warning that a large part of the word frequency in LDT may be due to the decision part and not to differences in word processing speed, even though there is evidence that the frequency effect is not completely absent from the word processing part (Allen et al., 2005). This once again points to the possibility that LDT data may say as much (and possibly more) about the task that is performed (binary decision) than about the process psycholinguists are interested in (the speed of word recognition). After all, in normal reading the job is not to decide the wordness of each letter string, but to activate the correct meanings of the letter strings. This discrepancy between reading and LDT is particularly worrying, given the low correlation we recently observed between lexical decision times and gaze durations to the same words in fluent text reading (Kuperman et al., 2012). Further complicating the picture is the finding that the correlation between RTs in LDT and gaze durations in reading is higher when the words are not part of continuous text but positioned in unconnected, neutral carrier sentences (Schilling et al., 1998). Clearly, more research is needed here to chart the commonalities among the tasks and the divergences.

A further sobering fact is the high internal noise we found for RTs. This was even true for the high-frequency words. Even though the high optimal model performance based on response accuracies (Figure 3) suggests that for these words RTs can be interpreted as the outcome of true word processing, we found no evidence that the internal/external RT noise ratio for these words was significantly lower than for low-frequency words. Our estimate of 8% for the optimal model performance with respect to RTs appears to apply irrespective of word frequency. This was true both in an analysis with a distinction between words with frequencies higher or lower than one per million, and in a more fine grained analysis attempting to predict the squared difference between trial-level RTs in the first and second pass (using a mixed model with participants and stimuli as crossed random factors and allowing for non-linearity via natural splines).

On a more general level, our analyses demonstrate considerable overlap between the LDT and psychophysical signal detection tasks. It appears that the degree of internal noise relative to the level of external noise is comparable between the two classes of tasks provided sensitivity is matched. The primary reason why the ratio is smaller in lexical decision than in representative psychophysical tasks (Neri, 2010) seems to be the higher visibility of the stimuli in lexical decision. It is relevant to this discussion that the inverse relation between internal noise and sensitivity we report for the lexical task (Figure 2A) has also occasionally been observed in some perceptual tasks (see Figure 4B in Burgess and Colborne, 1988) but not in others. As for the latter, Neri (2010) reported no correlation between sensitivity and internal noise for the datasets considered in his article (see also Gold et al., 1999). However, the range of sensitivity values spanned in these datasets was smaller than the one we report here for LDT (most data points in Neri (2010) fell below a value of 2 whereas our sensitivity data are mostly above 2, see the *y* axis in Figure 2A). This difference in range may account for the lack of correlation reported by Neri (2010), and points to the importance of establishing whether the relation between sensitivity and internal/external noise ratio represents a fundamental property of the human cognitive system that applies to a broader range of different choice paradigms or whether it presents different characteristics across cognitive tasks. Not just for this reason, but also for the purpose of generally becoming more aware of the importance and impact of internal in/consistency, we believe it is critical to take the current analyses to different areas of cognitive research.

The similarity of lexical decision to other signal detection tasks illustrates the utility of using mathematical models of lexical decision that include noise both at the RT and response choice level. Models of this kind are being developed (see in particular the drift diffusion model of Ratcliff et al., 2004; also see Norris and Kinoshita, 2012), but at present they do not provide the same flexibility of data-analysis as the linear models (e.g., Rigby and Stasinopoulos, 2005). It will be interesting to see to what extent these models will be able to simultaneously account for the usual factors influencing word processing and the degree of noise observed in the present study (for example with respect to the frequency curve, as shown in Figure 3).

To summarize, we have for the first time analyzed the level of internal noise associated with response choice and RTs in the LDT. The results show lower internal noise values for response choice than for RTs. Non-word choices and word choices for words with a frequency above 10 per million are especially consistent. The results for words with frequencies of less than 10 per million words indicate a substantial degree of guessing, seriously questioning the validity of RT data for these stimuli – at least with the LDT. An optimal response choice model could reach more than 90% accuracy for non-words and high-frequency words, whereas an optimal RT model would only explain about 8% of the trial-level data, irrespective of word frequency. It is important to keep these figures in mind when data are analyzed with linear models, because there is no way of directly estimating them in the usual single pass lexical decision experiment. It will also be interesting to understand the extent to which models that do not assume fixed response choices will be able to account for the present findings.

## Conclusion

We ran a signal detection analysis on the responses in a LDT (both response choices and RTs) to have a quantitative estimate of the noise in this task. Given that we found rather high levels of noise under some circumstances, these are the implications we see for researchers using LDT to investigate word processing:

1. LDT is a signal detection task with a rather high degree of noise, also in response choices, implying that not all word responses come from trials in which the participant recognized the stimulus as a known word. This is particularly the case for words known by less than 80–90% of the participants, and for participants who know less than 80–90% of the words. In these cases, rather high percentages of word responses seem to be guesses that turn into non-word responses when the block of stimuli is repeated.
2. Because of the noise in the response choices, RTs of “correct” responses should be treated cautiously if they come from conditions with more than 10% errors. This may be an issue, for instance, when data are compared across tasks.
3. If authors want to base their conclusions on RTs, they are advised to make sure the stimuli are known to their participants. Possible sources for this are the percentages known in the English Lexicon Project (Balota et al., 2007; 40,000 words) and the British Lexicon Project (Keuleers et al., 2012; 28,000 words). Another variable to take into account in this respect is the vocabulary size of the participants (Diependaele et al., 2012; Kuperman and Van Dyke, 2012).
4. The good performance for well-known words and for most non-words suggests that two response thresholds are used in LDT. This finding may be worthwhile to integrate in computational models of the task (Davis, 2010; Dufau et al., 2012).

## Conflict of Interest Statement

The authors declare that the research was conducted in the absence of any commercial or financial relationships that could be construed as a potential conflict of interest.

## Footnotes

^1^Output sensitivity *d*′ needs to be distinguished from input sensitivity $d_{in}^{\prime }$. It is the signal discriminability index derived from the actual performance, including internal noise. Hence, it is generally smaller than $d_{in}^{\prime }.$

^2^Following Neri (2010) the data of one participant are not shown in panel D because the RT-based noise estimates did not meet the criterion: $\frac{1}{5}\geq \times \geq 5$.

^3^The lower bound is given by the percentage of agreement α and the upper bound by the formula $\frac{1+\sqrt{2\alpha -1}}{2}.$

^4^This *within*-participant replication compares to an average *between*-participant replication of *R*^2^ = 0.02 ± 0.02 SD.

^5^Psycholinguists typically deal with the high level of noise in RTs by taking mean RTs across a group of participants (usually around 40). Rey and Courrieu (2010) reported that this practice indeed increases the reliability of the RT-values of the Dutch Lexicon Project to 84%. It is important to keep in mind, however, that this value represents the replicability of variance at the level of item RTs *averaged over a group of participants*. The present analysis shows that at the individual level, only some 8% of the variance is systematic variance. Especially in the context of computational models, it is critical to ask whether predictions should be made at the levels of average or individual data. In the latter case it may be more sensible to correlate the average performance over several runs of the model with the average performance of a group of participants.
